# PolyRound: polytope rounding for random sampling in metabolic networks

**DOI:** 10.1093/bioinformatics/btab552

**Published:** 2021-07-30

**Authors:** Axel Theorell, Johann F Jadebeck, Katharina Nöh, Jörg Stelling

**Affiliations:** Department of Biosystems Science and Engineering, SIB Swiss Institute of Bioinformatics, 4058 Basel, Switzerland; Institute of Bio- and Geosciences, IBG-1: Biotechnology, Forschungszentrum Jülich, 52425 Jülich, Germany; Institute of Bio- and Geosciences, IBG-1: Biotechnology, Forschungszentrum Jülich, 52425 Jülich, Germany; Computational Systems Biotechnology (AVT.CSB), RWTH Aachen University, 52062 Aachen, Germany; Institute of Bio- and Geosciences, IBG-1: Biotechnology, Forschungszentrum Jülich, 52425 Jülich, Germany; Department of Biosystems Science and Engineering, SIB Swiss Institute of Bioinformatics, 4058 Basel, Switzerland

## Abstract

**Summary:**

Random flux sampling is a powerful tool for the constraint-based analysis of metabolic networks. The most efficient sampling method relies on a rounding transform of the constraint polytope, but no available rounding implementation can round all relevant models. By removing redundant polytope constraints on the go, PolyRound simplifies the numerical problem and rounds all the 108 models in the BiGG database without parameter tuning, compared to ∼50% for the state-of-the-art implementation.

**Availability and implementation:**

The implementation is available on gitlab: https://gitlab.com/csb.ethz/PolyRound.

**Supplementary information:**

Supplementary data are available at *Bioinformatics* online.

## 1 Introduction

Random sampling of constraint-based models of metabolism is a powerful approach to characterize the potential behaviors of metabolic networks (Schellenberger and Palsson, 2009; Herrmann *et al.*, 2019). The field is actively developed, for example, with recent extensions to model inference (Theorell and Nöh, 2020). Algorithmically, Markov Chain Monte Carlo (MCMC) based coordinate hit-and-run with rounding (CHRR) (Haraldsdóttir *et al.*, 2017) showed superior performance in computational benchmarks (Herrmann *et al.*, 2019) and it is available in a highly efficient and modular implementation (Jadebeck *et al.*, 2021).

The relevant sampling space is a polytope *P*: a bounded set in $R^{n}$ constrained by hyperplanes. In constraint based models, *P* originates from stoichiometric reaction constraints and capacity constraints that give rise to equalities and inequalities. $P:=\{x\in R^{n}:A_{eq}x=b_{eq},A_{\mathrm{ineq}}x\leq b_{\mathrm{ineq}}\}$ with matrices $A_{eq}\in R^{m,n}$ and $A_{\mathrm{ineq}}\in R^{k,n}$, and vectors $b_{eq}\in R^{m}$ and $b_{\mathrm{ineq}}\in R^{k}$. For Hit-and-Run samplers, such as CHRR, the asymptotic mixing time (a common efficiency measure in MCMC) depends quadratically on the sandwiching ratio, the ratio of radii of the largest sphere contained in *P* and the smallest sphere containing *P* (Lovász and Vempala, 2006). Efficient random sampling therefore relies on an efficient ’rounding’ preprocessing step: it applies a linear transformation to make the polytope more spherical. In practice, sampling the rounded polytope converges within minutes, whereas sampling the unrounded polytope fails to converge in reasonable time.

Deterministic and stochastic algorithms for polytope rounding exist (Mangoubi and Vishnoi, 2019; Martino *et al.*, 2015). All current implementations of CHRR rely on deterministic search for the maximum volume ellipsoid (MVE) (Zhang and Gao, 2003); after rounding via a linear transform, the MVE equals the unit sphere. An implementation that handles polytopes formulated as *P* is interfaced from the CobraToolbox (CT) (Heirendt *et al.*, 2019). However, we found that it rounds only about half of the models in the BiGG repository (King *et al.*, 2016) due to numerical failures. Because this strongly limits the scope of models for random sampling, we provide PolyRound, an open source Python toolbox that uses a modified reformulation and rounding scheme optimized for robustness.

## 2 Materials and methods

### 2.1 Workflow

In a first step, PolyRound reformulates *P* in a form with only inequality constraints and embeds it in a space where it has non-zero hypervolume. With the null space matrix $N\in R^{l,n}$ of *A_eq_* (computed by SVD), we express *P* in the null space coordinates *u* = *Nx* + x_0_ (*x*_0_ is an arbitrary feasible point) using only inequality constraints. However, *P* may still have zero hyper volume, since the inequality constraints may contain the so-called 0-facets, directions in which the width of *P* is 0. Therefore, prior to reformulation, PolyRound computes all facet widths by sequentially locating the minimal and maximal feasible point in the direction orthogonal to each inequality constraint. This requires solving two linear programs (LPs) per constraint. If the width is smaller than a threshold (e.g. $10^{-7}$), the corresponding constraint is a de-facto equality constraint and is moved to the equality system. PolyRound also checks for redundant inequality constraints and removes these, using a third LP in which the right hand side of the constraint under investigation is relaxed. After all redundant constraints have been removed and 0-facet constraints have been refunctioned, the now smaller problem can be solved more accurately, so that new redundant constraints and 0-facets may be detected. Therefore, PolyRound iterates until no more changes of *P* are induced.

In a second step, PolyRound computes the MVE using the F2PD algorithm by Zhang and Gao (2003). The PolyRound MVE is an optimized python implementation of CT’s default routine Bounciness/Volume-and-Sampling (Haraldsdóttir *et al.*, 2017), including an iterative scheme that improves numerical stability (Supplementary Information S3).

### 2.2 Implementation

PolyRound is implemented in Python 3 and equipped with an easy-to-use command line interface. It reads constraint based models in SBML format (Hucka *et al.*, 2003) using COBRApy (Ebrahim *et al.*, 2013), and generic polytopes in plain HDF5 and csv representations. LPs are solved via optlang (Jensen *et al.*, 2017), enabling easy use of different solvers. To reproduce the benchmarks, see Supplementary Information.

## 3 Benchmarks

**Fig. 1.**
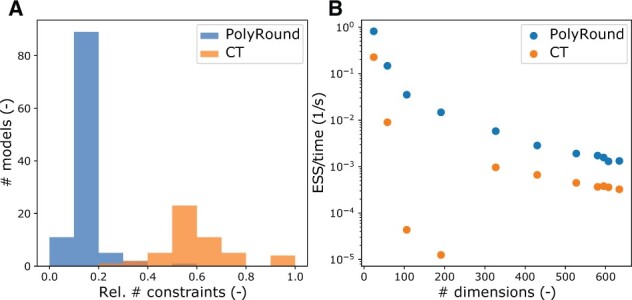
(**a**) Fraction of constraints after rounding (number of rows of the processed inequality matrix), relative to the number for unrounded BiGG models (number of rows of *A_ineq_*). Results for CT: best commit and default parameters. Due to failed rounding, the orange bars have ∼50% of the surface area of the blue bars. (**B**) ESS per time for a selection of models (Supplementary Table S1). The number of dimensions refers to the PolyRound processed models

We first assessed success in terms of obtaining a rounded polytope. PolyRound successfully rounded 100% of the 108 models in the BiGG database (King *et al.*, 2016) (Fig. 1A) , compared to at most 51% (Supplementary Fig. S1) for CT, using different parameters and versions. The performance difference is due to PolyRound’s primary invention, removal of redundant constraints: without it, PolyRound’s success rate dropped to 67% (see Supplementary Information). PolyRound and CT produced similar reductions in dimensionality, compared to the expected original dimensionality, but PolyRound achieved substantially larger reductions of the number of constraints (Fig. 1A, Supplementary Fig. S2), thus easing numerical computations in the rounding workflow. To validate that the polytopes generated by PolyRound yield efficient sampling, we collected 11 models spanning a range of sizes (Supplementary Table S1). The effective sample size (ESS) per time for uniform sampling with the HOPS library (Jadebeck *et al.*, 2021) was consistently higher for PolyRound than for CT (Fig. 1B).

## 4 Conclusion

PolyRound is an open-source, robust implementation of polytope rounding, which, by numerical craftsmanship, strongly widens the number of constraint based models for random sampling. Rounded models are maximally constraint reduced, which speeds up later computations.

## Supplementary Material

btab552_Supplementary_Data
