# Supplementary material for: PolyRound: polytope rounding for random sampling in metabolic networks
Source: Bioinformatics. 2021 Jul 30;38(2):566–7. doi: 10.1093/bioinformatics/btab552 (PMC8723145; doi:10.1093/bioinformatics/btab552)
Supplement: btab552_Supplementary_Data [file btab552_supplementary_data.zip › polyround_supplement_R2.pdf]

Supplementary Information for

PolyRound: Polytope Rounding for Random Sampling in Metabolic Networks

Axel Theorell [1,2,*], Johann F. Jadebeck [2,3], Katharina Nöh [2] and Jörg Stelling [1,*]

[1]Department of Biosystems Science and Engineering, and SIB Swiss Institute of Bioinformatics, ETH Zurich, 4058 Basel, Switzerland.

[2] Institute of Bio- and Geosciences, IBG-1: Biotechnology, Forschungszentrum Jülich, 52425 Jülich, Germany

[3] Computational Systems Biotechnology (AVT.CSB), RWTH Aachen University, 52062 Aachen, Germany.

[*]Correspondence to: joerg.stelling@bsse.ethz.ch

S.1 Benchmark Reproduction

This section describes the workflow of rounding all models in the BiGG database, which is used as a benchmark in the main text. A snapshot of the git repository and the Matlab and Python scripts that were used to produce the results is available at: `http://doi.org/10.5905/ethz-1007-383`.

1. All 108 models in the BiGG database (24.11.2020) were downloaded using the bigg-model-downloader (`https://github.com/qacwnfq/bigg-model-downloader`).
2. For PolyRound, the benchmark was executed with the PolyRound function *PolyRoundApi.simplify_transform_and_round*. PolyRound was at commit hash `commit 5db0b487e84ad404b74b0222510b76d8c2050586`.
3. For the CobraToolbox (CT), four attempts were made, using the CT function *sampleCbModel*, but with:
   (a) CT at the newest commit (Version v3.1, commit hash `92518352da3dc1f63b7ef781345786f5cf73034c`)
   (b) CT at the newest commit (Version v3.1, commit hash `92518352da3dc1f63b7ef781345786f5cf73034c`) and the extra parameter settings:
   $feasTol = 1e-9$, $optTol = 1e-9$.
   (c) CT at the older commit (No version tag, commit hash `c67a68b9e160d9da7ed97caa5026bf35a5ca3aa5`)
   (d) CT at the older commit (No version tag, commit hash `c67a68b9e160d9da7ed97caa5026bf35a5ca3aa5`) and the extra parameter settings:
   $feasTol = 1e-9$, $optTol = 1e-9$.

For both PolyRound and CT, Gurobi 9 was used as linear programming solver.

S.1.1 Specific Error Messages

Error messages produced when the rounding failed are provided for the 4 CT configurations (Fig. S.1). Detailed error messages for every model are provided in Supplementary Table 1. It also contains errors produced by PolyRound when redundant constraints are not removed. In the following, we comment on the error messages and contrast CT with PolyRound.

S.1.1.1 CT Error Messages

1. The three optimization errors *[gurobi] Dual optimality condition in solveCobraLP not satisfied*, *[gurobi] Primal optimality condition in solveCobraLP not satisfied* and *[gurobi] Optimality condition (1) in solveCobraLP not satisfied* refer to that the solution to a linear program returned by Gurobi does not fulfil the accuracy checks of CT. Inaccuracies in linear programs are handled differently in PolyRound, depending on whether they occur during simplification or rounding.

   - Simplification stage in PolyRound: Three linear programs per inequality constraint are solved to determine whether the constraint is superfluous or constitutes a zero width facet. At this stage, errors or inaccuracies by the LP solver may result in the rounded polytope no longer mapping back to the original polytope, which is unacceptable. Therefore, PolyRound implements strict checks, similar to CT; these checks were passed for all benchmarks.
   - Rounding stage in PolyRound: LPs are used to find the Chebyshev center, which is then used to rescale and center the polytope. In the same way as in CT, the maximum volume ellipsoid problem (MVE) is solved a number of times for a series of increasingly hard polytopes (see sec. S.3 for details). The last polytope is the actual (but scaled) polytope plus an additive regularization of 1e-9. If, for the first of these problems, the found Chebyshev center is not interior, a similar rounding problem is solved, in which the Chebyshev center is interior. With each solved MVE, the original polytope is rescaled, easing the solution of consecutive Chebyshev center and MVE problems for the series of polytopes. Because the rounding as such only is the construction of an affine linear bijective transform, inaccuracies in linear programming solutions are of no consequence as long as the algorithm converges and produces such a transform. To be sure that this is fulfilled for PolyRound, two checks are performed:

     (a) The affine mapping must have full rank once the algorithm terminates.

     (b) A strictly interior point must exist.

     Specifically for the testing of the affine mapping, PolyRound requires the smallest eigenvalue of the transformation to be larger than the most narrow allowed facet width (default is 1e-7) times a buffer of 1e-2 for numerical inaccuracy.

2. The errors *Input to SVD must not contain NaN or Inf* and *model.A must be a sparse double real matrix* occur when NaNs enter into the ellipsoid matrix while solving the MVE problem. This happens as a consequence of inversion of ill-conditioned matrices. As expanded in section S.1.1.2, this behavior is observed also in PolyRound, when removal of redundant constraints is omitted, indicating that regular PolyRound circumvents ill-conditioning by removing redundant constraints.

3. *x0 not interior* refers to the interior point provided to the rounding algorithm lying outside the polytope. PolyRound does not require the initial point in the rounding to be strictly interior (see section S.3 for details).

S.1.1.2 PolyRound with Redundant Constraints Error Messages

1. *array must not contain infs or NaNs* is caused by failing numerics during rounding, similar to point 2 in section S.1.1.1

2. *Rounding transformation not full dimensional* corresponds to failure of the check introduced in point (a) in the sub-enumeration in section S.1.1.1.

3. *Zero point not inside rounded polytope* corresponds to failure of the check introduced in point (b) in the sub-enumeration in section S.1.1.1.

4. *timeout* is an error that occurred on the server due to time out. The three timeouts only marginally affect the total success rate (68-70%).

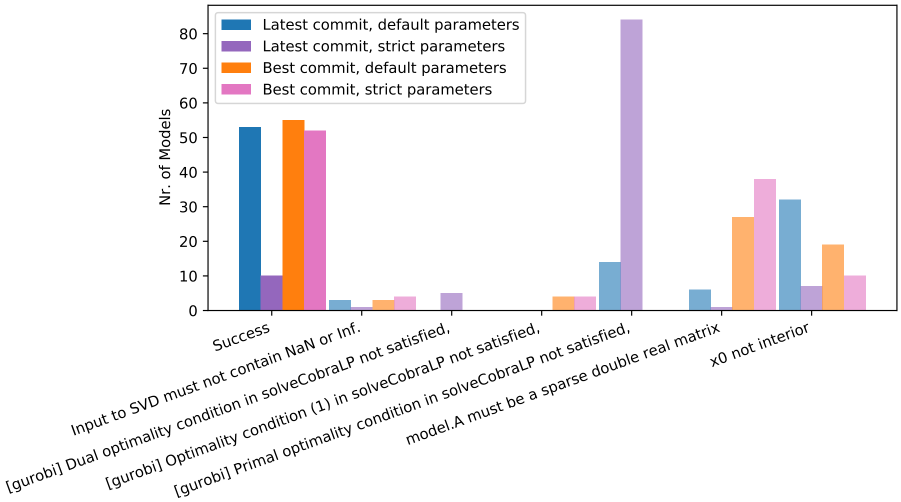

Figure S.1: The number of succeeded and failed models for the four CT configurations that correspond to (a)–(d) in the listing above. The x-tick labels are the specific error messages returned by CT. The error messages *[gurobi] Dual optimality condition (1) in solveCobraLP not satisfied*, *[gurobi] Optimality condition (1) in solveCobraLP not satisfied* and *[gurobi] Primal optimality condition (1) in solveCobraLP not satisfied* have been truncated, since the last part of the error message yielded a varying numerical value that obstructed classification.

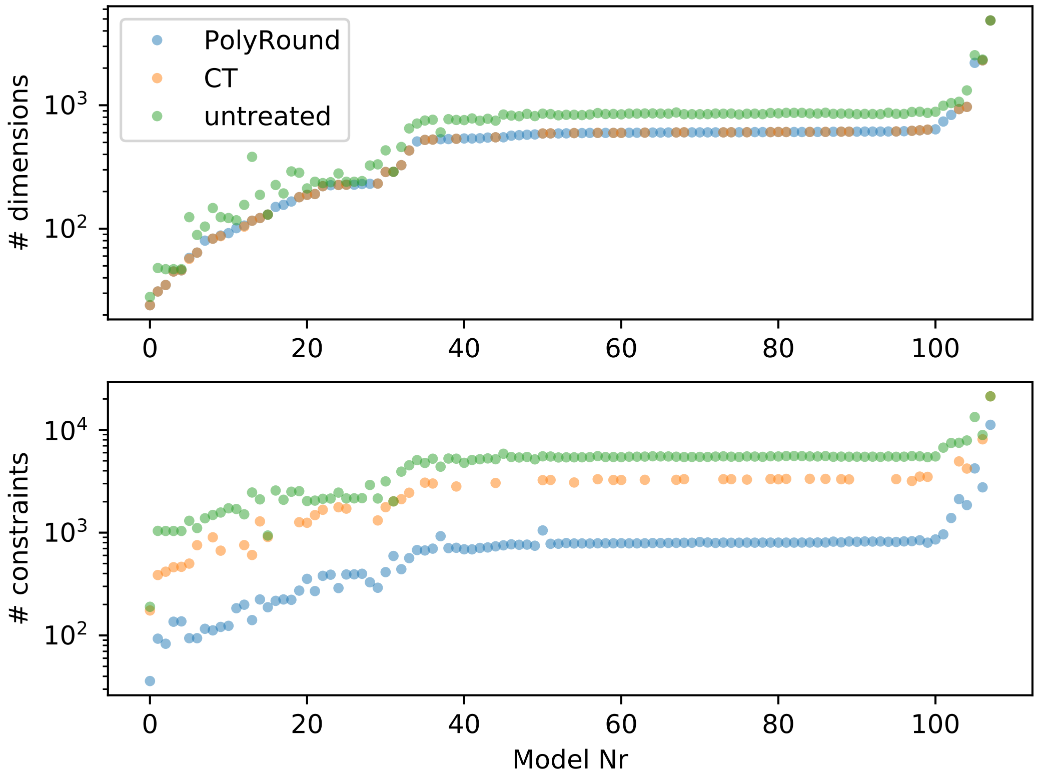

Figure S.2: Number of dimensions and constraints per model. Original represents the original model, where the number of dimensions is the number of columns of A_{eq} minus the rank of A_{eq}. Original number of constraints is the number of rows of A_{ineq}. After rounding, the number of dimensions and constraints is the number of columns and rows of the processed inequality matrix. Results for CT are shown for the best commit and default parameters. The underlying data is available in the file `Supplementary Table 1.csv`.

Model name	Nr. of dimensions
e coli core	24
iLJ478	58
iNF517	106
iNJ661	191
IPC815	327
iYL1228	430
iS 1188	527
iJO1366	580
iECED1 1282	595
iECW 1372	607
iML1515	634

Table S.1: The models used in the sampling benchmark (Fig. 1B main text) and their dimensions after PolyRound processing.

S.2 Sampling Reproduction

The sampling results in Fig. 1B in the main manuscript were generated with the Highly-Optimized Polytope Sampling library HOPS (`https://github.com/modsim/hops`, tag `v1.10`), using the application *SamplingUniformTarget*. The polytopes were interfaced between the rounding software PolyRound and HOPS via .csv files. The rounded CT polytopes were generated with CT configuration (a) described in the listing in section S.1.

S.3 Iterative Rounding Scheme

After simplifying and transforming the polytope, PolyRound applies the F2PD rounding algorithm iteratively until the polytope is sufficiently round. This rounding scheme largely follows the one in CT. However, it includes a small alteration to accommodate for the specific numerical problems originating from scaling in the early iterations of the algorithm. We first show the general scheme and then discuss the alteration.

As in the main manuscript, we start with the general (in this case already simplified) polytope $P := \{x \in \mathcal{R}^n : A_{eq}x = b_{eq}, A_{ineq}x \leq b_{ineq}\}$ with matrices $A_{eq} \in \mathcal{R}^{m,n}$ and $A_{ineq} \in \mathcal{R}^{k,n}$, and vectors $b_{eq} \in \mathcal{R}^m$ and $b_{ineq} \in \mathcal{R}^k$. After transformation, using a (generally non-square) transformation matrix $T_{rect} \in \mathcal{R}^{n,l}$, we get a new polytope with only inequality constraints $P_0 := \{\nu \in \mathcal{R}^l : A_0\nu \leq b_0\}$ with $A_0 = A_{ineq}T_{rect} \in \mathcal{R}^{k,l}$, and vectors $b_0 = b_{ineq} \in \mathcal{R}^k$. It is assumed that the new polytope has an interior point ν_0. Algorithm 1 is executed on the transformed polytope and produces a rounded polytope, expressed by the rounding transformation matrix, $T \in \mathcal{R}^{l,l}$, and the shift vector, $t \in \mathcal{R}^l$. Inside Algorithm 1, T, t, A_0 and b_0 are given indices that indicate iteration number.

After a successful run of Algorithm 1, given that it converged after n_i iterations, it holds true that

$$T = T_0 \cdot \ldots \cdot T_{n_i}, \quad (1)$$

$$t = t_0 + T_0t_1 + T_0T_1t_2 + \ldots + T_0 \cdot \ldots \cdot T_{n_i-1}t_{n_i} \quad (2)$$

$$A_{n_i+1} = A_0T_0 \cdot \ldots \cdot T_{n_i} = A_0T, \quad (3)$$

$$b_{n_i+1} = b_0 - A_0t_0 - A_0T_0t_1 - \ldots - A_0T_0 \cdot \ldots \cdot T_{n_i-1}t_{n_i}t_{n_i} = b_0 - A_0t \quad (4)$$

Thus, given that t is finite and T is invertible (guaranteed in PolyRound), we have an affine linear bijection between the transformed space P_0 and the rounded space $P_R := \{\nu \in \mathcal{R}^l : A_{n_i+1}\nu \leq b_{n_i+1}\}$. Thus, the interior points in one map to the interior points in the other.

Note that each execution of F2PD (line 10, Algorithm 1) yields a rounded polytope. Indeed, if it were possible to solve the problem with exact arithmetics, the iterative scheme would be unnecessary and rounding would result from a single execution of F2PD. Since we round using finite precision and badly conditioned matrices may cause F2PD to fail, the non-zero regularization λ is introduced. A non-zero regularization means that, for example in the first iteration, instead of rounding exactly the polytope P_0, a slightly different, regularized polytope is rounded and this rounding transform is applied on P_0. A consequence of F2PD rounding the polytope in every iteration is that, how *round* the final polytope is, is entirely determined by the last iteration, in which the polytope with the smallest regularization, and thus the one most similar to the actual polytope, is rounded. The function of the

Algorithm 1: Original iterative rounding scheme (not new in PolyRound)

Input: $A_0 \in \mathcal{R}^{m,l}$, $b_0 \in \mathcal{R}^m$

// T and t is a matrix and a vector that transform P_0 to the rounded polytope

Output: T, t

// C is a function that finds the Chebyshev Center (an interior point) of a polytope

$\nu_0 \leftarrow \mathrm{C}(A_0, b_0)$

$\lambda \leftarrow 10^{-3}$

$t \leftarrow 0$

// $\mathcal{I}$ is the identity matrix of dimension l

$T \leftarrow \mathcal{I}$

$i \leftarrow 0$

while *not converged* **do**

  // Move the polytope to be centered around ν

  $b_i' \leftarrow b_i - A_i \nu_i$

  // Normalize the polytope so that all elements of the right hand side are 1 (does not alter polytope shape)

  $A_i' \leftarrow \text{row-divide}(A_i, b_i')$

  $b_i'' \leftarrow \text{element-divide}(b_i', b_i') = [1 \dots 1]^T \in \mathcal{R}^k$

  // Perform F2PD on the normalized system, under the regularization λ. γ_i contains information about the execution, such as convergence.

  $t_i, T_i, \gamma_i \leftarrow \text{F2PD}(A_i', b_i'', \lambda)$

  // Update the transformation and the polytope

  $t \leftarrow t + T t_i$

  $T \leftarrow T T_i$

  $b_{i+1} \leftarrow b_i - A_i t_i$

  $A_{i+1} \leftarrow A_i T_i$

  $\nu_{i+1} \leftarrow \mathrm{C}(A_{i+1}, b_{i+1})$

  $\lambda \leftarrow \lambda / 10$

  $i \leftarrow i + 1$

  // Check convergence criteria that consider aspects of λ, γ_i and A_{i+1}

  Check convergence

early iterations, with larger regularization, is only to make the rounding problem easier, so that the last iteration can complete without numerical failures. The fact that the early iterations do not affect the roundness of the final polytope is used to make two improvements in PolyRound (Algorithm 2). First of all, even though the simplified polytope P is guaranteed to have an interior point (by explicit tests), in finite precision, we do not know for sure that the linear program searching for an interior point (line 1, Algorithm 2) will actually find an interior point in P_0. As a remedy, lines 8-9 in Algorithm 2 introduce an alteration of the polytope, Δ_b, which guarantees that the found ν_i is interior and that the distance to all constraints is at least ϵ_b (default in PolyRound: 10^{-7}). Similarly, a second problem is that, when normalizing the matrix A_i (line 12, Algorithm 2), if the elements of b_i' span too many orders of magnitude, the resulting A_i' matrix may get ill-conditioned. Again, as a remedy, lines 10-11 Algorithm 2 introduce an alteration Δ_s, which guarantees that the ratio between the largest and smallest elements of b_i' is no larger than ϵ_s (default in PolyRound: 10^8). Since the roundness of the final polytope is determined entirely by the last iteration, the impact of Δ_b and Δ_s on the roundness is zero, in case they go to zero before the algorithm terminates. Therefore, PolyRound requires that Δ_b and Δ_s are zero in the last iteration for the rounding to be valid. At this point, we do not have a mathematical proof that Δ_b and Δ_s always go to zero with increasing iterations. However, given their role in Algorithm 2, this seems plausible. Hitherto, they went to zero in all cases we have encountered.

Algorithm 2: Polyround iterative rounding scheme

Input: $A_0 \in \mathcal{R}^{m,l}$, $b_0 \in \mathcal{R}^m$, $\epsilon_b > 0$, $\epsilon_s > 0$

// T and t is a matrix and a vector that transform P_0 to the rounded polytope

Output: T, t

// C is a function that finds the Chebyshev Center (an interior point) of a polytope

$\nu_0 \leftarrow \mathrm{C}(A_0, b_0)$

$\lambda \leftarrow 10^{-3}$

$t \leftarrow 0$

// $\mathcal{I}$ is the identity matrix of dimension l

$T \leftarrow \mathcal{I}$

$i \leftarrow 0$

while *not converged* **do**

// Move the polytope to be centered around ν_i

$b'_i \leftarrow b_i - A_i\nu_i$

// Get boundary alteration sizes Δ_b and alter the polytope so that all elements of b'_i are larger than 0 (element wise maximum)

$\Delta_b \leftarrow b'_i - \max(b'_i\epsilon_b)$

$b'_i \leftarrow \max(b'_i, \epsilon_b)$

// Get scaling alteration sizes Δ_s and alter the polytope so that the range of b'_i is smaller than ϵ_s

$\Delta_s \leftarrow b'_i - \min(b'_i, \min(b'_i) \cdot \epsilon_s)$

$b'_i \leftarrow \min(b'_i, \min(b'_i) \cdot \epsilon_s)$

// Normalize the polytope so that all elements of the right hand side are 1 (does not alter polytope shape)

$A'_i \leftarrow \text{row-divide}(A_i, b'_i)$

$b''_i \leftarrow \text{element-divide}(b'_i, b'_i) = [1 \ldots 1]^T \in \mathcal{R}^k$

// Perform F2PD on the normalized system, under the regularization λ. γ_i contains information about the execution, such as convergence.

$t_i, T_i, \gamma_i \leftarrow \mathrm{F2PD}(A'_i, b''_i, \lambda)$

// Update the transformation and the polytope

$t \leftarrow t + Tt_i$

$T \leftarrow TT_i$

$b_{i+1} \leftarrow b_i - A_i t_i$

$A_{i+1} \leftarrow A_i T_i$

$\nu_{i+1} \leftarrow \mathrm{C}(A_{i+1}, b_{i+1})$

$\lambda \leftarrow \lambda/10$

$i \leftarrow i + 1$

// Check convergence criteria that considers aspects of λ, γ_i, A_{i+1}, Δ_b and Δ_s

Check convergence

S.4 Development Notes

During development of PolyRound, the BIGG models Ecoli_core, iNJ661, iECW_1372, and Recon3D were used as test models. Development with these models led us to three important realizations:

1. We do not need to have a strictly interior point at the beginning of rounding (see section S.3).
2. With iNJ661, the linear programming solver sometimes got stuck. The issue was resolved by automatically detecting when it gets stuck and rerunning the solver from a new starting point.
3. Solving as many linear programs in sparse space as possible is critical before reducing the polytope to have only inequality constraints with the SVD.

In early development, PolyRound relied on Gurobi directly through its python interface gurobipy. To ease this dependency on commercial software, PolyRound was updated to use optlang to interface to multiple LP solvers. With this change, we could rerun our benchmarks with the LP solver GLPK. However, using GLPK, we sometimes encountered numerical problems related to the scaling performed on line 8 of Algorithm 1, which led us to implement the improved scaling control in Algorithm 2 (section S.3).

S.5 Rounding with GLPK

Since PolyRound uses optlang to interface to multiple LP solvers, it is straightforward to change the solver backend from the commercial solver Gurobi (default if Gurobi is installed) to the open source solver GLPK. Rerunning the BiGG database with GLPK as LP backend, 94% of the models were rounded successfully. Seven models failed because they violated the PolyRound default timeout (see Supplementary Table 1), which does not allow a single LP to run for longer than 10 minutes. It is to be expected that the open source GLPK solver is slower than the commercial Gurobi solver. Overall, we conclude that rounding results are reproducible with a different solver.
